# Hmgb1-IL-23-IL-17-IL-6-Stat3 Axis Promotes Tumor Growth in Murine Models of Melanoma

**DOI:** 10.1155/2013/713859

**Published:** 2013-12-28

**Authors:** Qiu Tang, Jian Li, Hongfei Zhu, Pan Li, Zhenwei Zou, Yin Xiao

**Affiliations:** ^1^Department of Oncology, The Central Hospital of Wuhan, Tongji Medical College, Huazhong University of Science and Technology, 26 Wuhan Shengli Road, Wuhan 430014, Hubei, China; ^2^Department of Oncology, Wuhan General Hospital of Guangzhou Command, People's Liberation Army, China; ^3^Department of Anesthesiology, Hospital of Stomatology, Wuhan University, Wuhan, China; ^4^Department of Immunology, Tongji Medical College, Huazhong University of Science and Technology, Wuhan, China; ^5^Department of Oncology, Wuhan Union Hospital, Wuhan, China

## Abstract

In order to understand how tumor cells can escape immune surveillance mechanisms and thus develop antitumor therapies, it is critically important to investigate the mechanisms by which the immune system interacts with the tumor microenvironment. In our current study, IL-17 deficiency results in reduced melanoma tumor size, diminished numbers of proliferating cells and blood vessels, and decreased percentage of CD11b^+^Gr-1^+^ MDSCs in tumor tissues. IL-17 promotes IL-6 induction and Stat3 activation. Treatment of Stat3 inhibitor WP1066 in B16-F10 tumor cells inoculated wild-type mice inhibits tumor growth. Additional administration of recombinant IL-6 into B16-F10 tumor-bearing IL-17^−/−^ mice results in markedly increased tumor size and p-Stat3 expression, whereas additional recombinant IL-17 administration into B16-F10 tumor-bearing wild-type mice treated with anti-IL-6 mAb does not significantly alter the tumor growth and p-Stat3 expression. In our further study, blockade of Hmgb1-RAGE pathway inhibits melanoma tumor growth and reduces production of IL-23 and IL-17. All these data suggest that Hmgb1-IL-23-IL-17-IL-6-Stat3 axis plays a pivotal role in tumor development in murine models of melanoma, and blocking any portion of this axis will attenuate melanoma tumor growth.

## 1. Introduction

In order to understand how tumor cells can escape immune surveillance mechanisms and thus develop antitumor therapies, it is critically important to investigate the mechanisms by which the immune system interacts with the tumor microenvironment. The tumor microenvironment potently inhibits immune responses against tumor cells through various soluble mediators and mechanisms [1–3].

IL-17 is an inflammatory cytokine which plays an important role in the regulation of leukocyte migration in inflammatory reaction [4–8]. The role of IL-17 in inflammatory and autoimmune diseases has been studied extensively [9, 10]. Until now, the role of IL-17 in tumor development is controversial. Recent reports showed that tumor growth was increased in IL-17 deficiency mice and the mechanism was associated with NK cells [11, 12]. Some other reports indicated inhibited tumor growth in IL-17 deficiency mice due to the increased myeloid-derived suppressor cells (MDSCs) infiltration and IL-6 production in tumors [1, 13]. High-mobility group box 1 (Hmgb1) is an evolutionarily conserved, chromatin-binding protein that has been implicated in several disease states including sepsis, arthritis, ischemia-reperfusion injury, and cancer [14–16]. Cancer cells that have undergone necrotic cell death can release Hmgb1 into the local microenvironment. Hmgb1 can induce chronic inflammatory-reparative responses and lead to tumor cell expansion and metastases. Hmgb1 is also actively secreted by inflammatory cells, acting as an endogenous danger signal and binding with high affinity to several receptors including TLR2, TLR4, TLR9, and RAGE [14]. Furthermore, report from Akirav et al. suggested association of RAGE expression with increased levels of IL-17 production [17]. However, the cellular and molecular immune mechanisms of Hmgb1 in the development of tumor remain elusive. How the specific innate immune receptor for Hmgb1 induces proinflammatory cytokines and how these cytokines prime the subsequent innate immune response are completely unclear.

In this study, we demonstrate that Hmgb1 stimulates the production of IL-23 in a RAGE-dependent manner. IL-23 promotes the expression of IL-17 which is mainly generated by *γδ* T cells. IL-17 then promotes tumor growth through IL-6 induction, which in turn activates Stat3 in tumors. Thus, the Hmgb1-IL-23-IL-17-IL-6-Stat3 axis contributes to tumor development in murine models of melanoma.

## 2. Materials and Methods

### 2.1. Mice

Wild-type C57BL/6 mice were from the Center of Experimental Animals, Tongji Medical College of Huazhong Science and Technology University, China. IL-17^−/−^ mice on C57BL/6 background and RAGE^−/−^ mice were purchased from Jackson Laboratory (Bar Harbor, ME, USA). All the mice were housed in specific pathogen-free facility with regular food and water ad libitum. Experiments were approved by the Institutional Animal Care and Use Committee at Tongji Medical College (Wuhan, China).

Transplanted tumor models: protocols for inoculation and measurement of tumors in transplanted tumor models were reported previously [18]. Mouse melanoma cell line B16-F10 was purchased from the American Type Culture Collection. Tumor cells were injected s.c. in mice, and tumor growth was monitored every 3 days. Tumor sizes were calculated with the following formula: tumor size = *L* × *S* × *H* × *π*/6 (*L*, long diameter; *S*, short diameter; *H*, height) [19].

To examine the effect of IL-17, IL-23, and IL-6 on tumor growth, mice were treated i.v. with adenovirus (Ad)-encoding GFP or mouse IL-17A (Ad-IL-17), IL-6 (Ad-IL-6), or IL-23 (Ad-IL-23) (10^9^ PFU/mouse) as described previously [20]. The recombinant adenoviruses used for mouse IL-17A, IL-23, or IL-6 overexpression were generated using the AdEasy Adenoviral Vector System (Stratagene, La Jolla, CA) in AD-293 cells according to manufacturer's instructions [21]. Two days later, the mice were inoculated with B16-F10 tumor cells.

To examine the effect of neutralizing IL-17 or IL-23 or IL-6 or *γδ* TCR on tumor growth, mice were inoculated s.c. with B16-F10 tumor cells and treated i.p. with 100 *μ*g/mouse of normal rat IgG or rat anti-mouse-IL-17 mAb (Biolegend, USA), -IL-23, -IL-6 (eBioscience, USA), and -*γδ* TCR, -NKG2D mAb (ATCC, Manassas, VA, USA) on days 0 (the day of tumor inoculation), 1, 6, 10, and 14 [19]. For inhibition of p-Stat3, mice were injected by oral gavage with WP1066 (Santa cruz biotechnology, Inc.) at 40 mg/kg in a mixture of 20 parts DMSO to 80 parts polyethylene glycol 300 (Sigma-Aldrich, Tokyo, Japan) once per day (5 days on and 2 days off) as described elsewhere [22, 23]. For inhibition of Hmgb1, mice were treated i.p. with 10 mg/mouse glycyrrhizin (TCI, Shanghai, China) on days 0 (the day of tumor inoculation), 2, 5, 10, and 15 [24].

To examine effects of MDSCs on tumor growth, purified MDSCs (2 × 10^6^/mouse) were coinjected s.c. with B16 melanoma cell (1 × 10^6^/mouse) in wild-type mice and tumor growth was monitored [19].

### 2.2. Immunohistochemical Analysis

B16-F10 tumor cells were inoculated into mice. After 2 weeks, tumor tissues were harvested from the mice. 5 *μ*m sections of flash-frozen tumor specimens were fixed in acetone, permeabilized with methanol, stained with antibodies specific to CD8 or anti-proliferating cell nuclear Ag (PCNA) or tyrosine-phospho-Stat3 (p-Stat3) (Santa Cruz Biotechnology, Inc.), and detected with secondary antibodies conjugated Alexa Fluor 488 (Invitrogen), as previously described [25]. Pictures were taken microscopically with a digital camera (Olympus, Melville, NY). Blood vessels were counted under light microscopy at ×200. Positive cells were counted in 10 fields of each group. Average numbers of positive cells per field were calculated and analyzed statistically.

### 2.3. Flow Cytometry Analysis

To examine tumor-infiltrating cells, tumor tissues from mice inoculated with B16 tumor cells 2 weeks ago were cut into small pieces and digested in RPMI 1640 medium containing collagenase D (2 mg/mL) (Sigma-Aldrich, St. Louis, MO), Dnase I (50 *μ*g/mL), and 10% FCS. To detect MDSCs, cell suspensions of tumors and spleens or blood leukocytes were stained with Alexa488-labeled CD11b and allophycocyanin-labeled Gr-1 Abs (BD Biosciences). For intracellular cytokine staining, lymphocytes were stimulated with plate-bound anti-CD3 and anti-CD28 mAb (BD Bioscience) in 96-well flat-bottomed plates for 6 h. For IL-17-producing analysis, lymphocytes were stained with anti-CD4, -CD8, -*γδ* TCR, and -IL-17 mAb (BD Biosciences) according to manufacturer's instructions. Data were acquired on a FACSCalibur (BD Bioscience) and analyzed using CellQuest software (BD Bioscience).

### 2.4. Tunel Assay

Apoptotic cells were detected by TUNEL assay as described previously [18, 19]. Briefly, tumor samples from mice inoculated with B16 tumor cells 2 weeks ago were fixed in 10% formalin, and sections were made. TUNEL assay was performed using a commercial apoptosis detection kit according to the manufacturer's instructions (Promega, Madison, WI). Sections were counterstained with DAPI and photographed microscopically with a ×10 objective. The number of apoptotic cells was counted, and results from 10 fields of each group were calculated for statistical analysis.

### 2.5. Realtime Quantitative RT-PCR

Total RNA was extracted from cultured cells or tissues using Trizol (Invitrogen, Carlsbad, CA) and reverse transcribed into cDNA using the PrimeScript RT reagent kit (Takara Biotechnology, Dalian, China) according to the manufacturer's instructions. mRNA levels of target genes were quantified using SYBR Green Master Mix (Takara Biotechnology, Dalian, China) with ABI PRISM 7900 Sequence Detector system (Applied Biosystems, Foster City, CA). Each reaction was performed in duplicate, and changes in relative gene expression normalized to *β*-actin levels were determined using the relative threshold cycle method. Primer sequences were shown in Supplemental Table 1 in Supplementary Material available online at http://dx.doi.org/10.1155/2013/713859.

### 2.6. Western Blot

The protein level of p-Stat3 was determined by Western blot using primary anti-p-Stat3 (Abnova, Taipei, Taiwan). Protein extracted from cells or tissue was separated on 10% SDS-polyacrylamide electrophoresis gels and transferred to nitrocellulose membranes (Pierce, Rockford, IL). After being blocked with 5% nonfat milk in TBS for 3 hours, the membranes were incubated with indicated primary antibodies (0.2 *μ*g/mL) at 4°C overnight, followed by incubation with HRP-conjugated secondary antibody (1 : 5000) for 3 hours. *β*-Actin was used as a loading control for comparison between samples.

### 2.7. ELISA

Signal-cell suspensions prepared from B16 tumors harvested from wild-type mice and IL-17^−/−^ mice were cultured *in vitro* overnight. IL-6 concentrations in culture supernatants were measured using ELISA kits from R&D System. For *in vitro* analysis, 10^6^/mL B16 cells were stimulated for 24 h with 10 ng/mL of recombinant IL-17, and then IL-6 concentrations were measured in culture supernatants [13].

### 2.8. Statistical Analysis

All data were presented as means ± SEM. The two-tailed Student's *t*-test was applied for statistical analysis with *P* < 0.05 being considered statistically significant. Data were analyzed using Prism software (GraphPad Software, Inc.).

## 3. Results

### 3.1. Tumor Growth Is Inhibited in IL-17^−/−^ Mice

**Figure 1:**
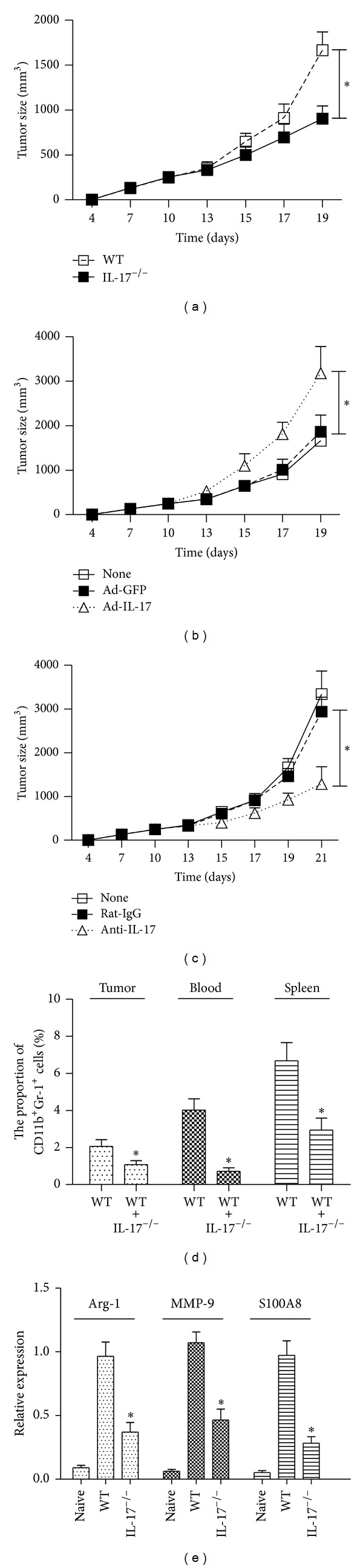
A defect in IL-17 inhibits tumor development. Mice were inoculated s.c. with tumor cells, and tumor sizes were monitored. (a) Wild-type mice and IL-17^−/−^ mice were inoculated with B16-F10 tumor cells (*n* = 5). (b) Wild-type mice were treated i.v. with Ad-IL-17 or Ad-GFP (10^9^ PFU/mouse) or left untreated (none). Two days later, the mice were inoculated with B16-F10 tumor cells, and tumor growth was monitored (*n* = 5). (c) Wild-type mice were inoculated with B16-F10 tumor cells and injected i.p. with normal rat-IgG or a rat anti-mouse IL-17 mAb (100 *μ*g/mouse) on days 0, 1, 6, 10, and 14 (*n* = 5). Control mice were left untreated (none). (d) CD11b^+^Gr-1^+^ MDSCs in the spleen, blood, and tumor samples from tumor-bearing mice inoculated with B16-F10 tumor cell 2 weeks ago were analyzed by FACS (*n* = 5). (e) MDSCs were purified from spleens of B16 tumor-bearing mice and stimulated overnight with LPS. CD11b^+^ cells from naïve tumor-free mice were treated with LPS and served as controls. mRNA levels were determined by realtime RT-PCR and normalized to *β*-actin in each sample (*n* = 5). The data show means ± SEM of tumor size and are representative of three independent experiments. *P < 0.05.

To examine the effect of IL-17 on melanoma growth, wild-type mice and IL-17^−/−^ mice were inoculated s.c. with melanoma cell line B16-F10 (1 × 10^6^/mouse), and tumor growth was monitored. Results showed that growth of melanoma cell line B16-F10 was significantly inhibited in IL-17^−/−^ mice compared with wild-type mice (Figure 1(a)). To further determine whether IL-17 promoted tumor growth, wild-type mice were injected i.v. with Ad-IL-17 or Ad-GFP (10^9^ PFU/mouse) and then inoculated with B16-F10 tumor cells. Results showed that the treatment with Ad-IL-17 significantly increased tumor growth compared with control mice that were treated with Ad-GFP or left untreated (Figure 1(b)). In contrast, wild-type mice with a neutralizing anti-IL-17 Ab treatment significantly inhibited the growth of B16-F10 tumors compared with controls that were treated with rat IgG (Figure 1(c)).

To examine effects of IL-17 deficiency on tumors, tumor tissues from tumor-bearing mice were collected and subjected to analysis. Results showed that the number of proliferating cells in tumors, which were stained with PCNA Ab, was significantly reduced in IL-17^−/−^ mice compared with wild-type animals (Supplementary Figure 1(a)). In contrast, the number of apoptotic cells, which were detected by TUNEL assay, was significantly increased in IL-17^−/−^ mice (Supplementary Figure 1(b)). The immunity at tumor sites is important for the fate of tumors, and the infiltration of T cells is closely associated with prognosis [26, 27]. We found that the infiltration of CD8^+^ T cells in tumors, which are the major effector cells for tumor rejection, was significantly increased in IL-17^−/−^ mice (Supplementary Figure 1(c)).

An increased number of MDSCs in spleen, blood, and tumors are a hallmark of major immunological abnormalities in cancer patients and tumor-bearing animals [28, 29]. MDSCs are considered as an immature form of myeloid cells, which are mostly identified as CD11b and Gr-1 double-positive cells in mice [30]. We found that the percentage of CD11b^+^Gr-1^+^ MDSCs in the spleen, blood, and tumors of IL-17^−/−^ mice was significantly reduced compared with wild-type controls (Figure 1(d)). The tumor-promoting function of MDSC is associated with increased activities of Arg-1, MMP9, and S100A8 [30]. In our experiment, MDSCs were purified from spleens of tumor-bearing mice and stimulated with LPS *in vitro* for overnight. The results showed that MDSCs from IL-17^−/−^ tumor-bearing mice expressed lower levels of Arg-1, MMP9, and S100A8 than those from wild-type tumor-bearing mice (Figure 1(e)).

Previous paper indicated that tumor cells overexpressing IL-17 significantly promote new vessel growth into the tumor tissue [7]. We also surveyed the vascular density in tumor tissues obtained from wild-type and IL-17^−/−^ mice. Immunohistochemical analysis revealed that the mean numbers of blood vessels in the tumor tissues were markedly decreased in IL-17^−/−^ mice (Supplementary Figure 1(d)).

### 3.2. Depletion of *γδ* T Cells Inhibited Tumor Growth

**Figure 2:**
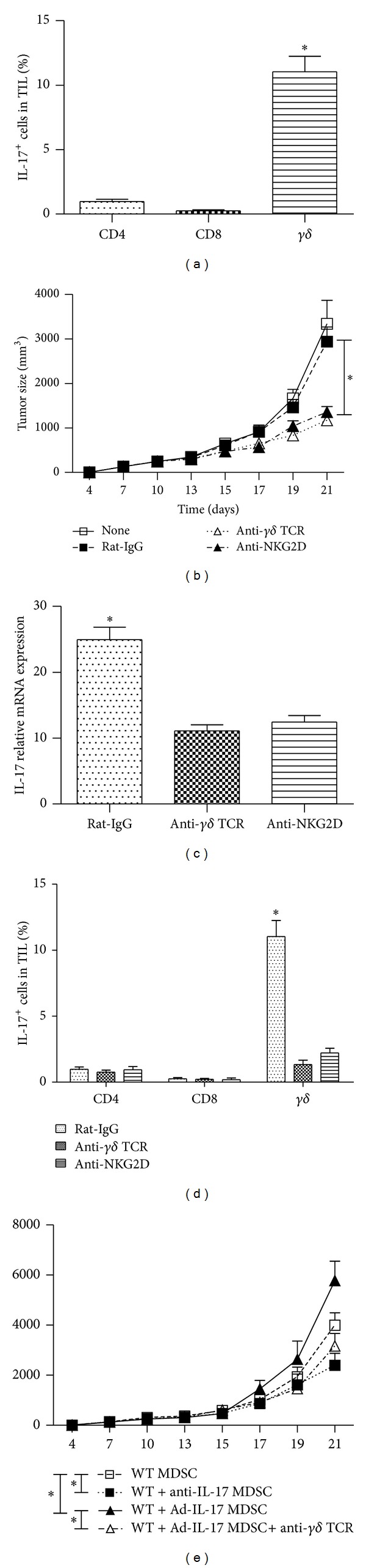
IL-17 is predominantly produced by *γδ* T cells, and depletion of *γδ* T cells inhibited tumor growth. TIL were collected from B16-F10 tumor tissues 2 weeks after B16-F10 tumor cell inoculation. IL-17-producing cells in each T-cell subset were detected by intracellular staining assay. (a) The percentage of cells producing IL-17 (*n* = 5). (b) Wild-type mice were inoculated with B16-F10 tumor cells and injected i.p. with normal rat-IgG or a rat anti-mouse *γδ* TCR mAb or anti-mouse NKG2D mAb (100 *μ*g/mouse) on days 0, 1, 6, 10, and 14 (*n* = 5). Then tumor sizes were monitored. (c) IL-17 relative mRNA expression in tumor tissues from B16-F10 tumor-bearing mice treated with rat-IgG, -*γδ* TCR, or -NKG2D mAb (*n* = 5). (d) The percentage of cells producing IL-17 in control, anti-*γδ* TCR, and anti-NKG2D treated group (*n* = 5). (e) Tumor growth in B16 tumor cells inoculated mice from WT MDSC, WT + anti-IL-17 MDSC, WT + Ad-IL-17 MDSC, and WT + Ad-IL-17 MDSC + anti-*γδ* TCR groups (*n* = 5). The data show means ± SEM of tumor size and are representative of three independent experiments. *P < 0.05.

To identify the cellular source of IL-17 production during tumor development, we prepared TIL from tumor-bearing mice 2 weeks after B16-F10 inoculation in wild-type mice. IL-17 production by distinct subsets of lymphocytes was analyzed by intracellular cytokine staining assay after stimulation with immobilized anti-CD3 and anti-CD28 mAb *in vitro*. We found that IL-17 was predominantly produced by *γδ* T cells rather than CD4^+^ T cells or CD8^+^ T cells infiltrated into the tumor tissues (Figure 2(a)).

Previous paper indicated that NKG2D expression was detected on tumor-infiltrating *γδ* T cells [1]. We added anti-*γδ* TCR or anti-NKG2D mAb into B16-F10 tumor-bearing mice. Results showed that growth of melanoma cell line B16-F10 in wild-type mice was significantly inhibited with treatment of anti-*γδ* TCR or anti-NKG2D mAb compared with treatment of rat-IgG or left untreated (Figure 2(b)). The number of proliferating cells and mean blood vessels and the percentage of CD11b^+^Gr-1^+^ MDSCs in tumor tissues were also significantly reduced with anti-*γδ* TCR or anti-NKG2D mAb administration (Supplementary Figures  2(a)–2(c)). At the same time, IL-17 production was profoundly blockade by anti-*γδ* TCR or anti-NKG2D mAb (Figure 2(c)). And the reduced IL-17 production was due to the decreased IL-17-producing *γδ* T cells (Figure 2(d)). These findings suggest that *γδ* TCR engagement is essential for IL-17 production by tumor-infiltrating *γδ* T cells within the tumor microenvironment, and NKG2D enhances these effects.

Furthermore, we investigated the contribution of MDSC and *γδ* T cells to tumorigenic effects. Results showed a reduced tumor-promoting effect of MDSCs from anti-IL-17 treated B16 tumor-bearing mice compared with that from wild-type counterparts. In contrast, MDSCs from wild-type tumor-bearing mice that were treated with Ad-IL-17 promoted tumor growth to a significant greater extent than those from control wild-type tumor-bearing mice. And the increased tumor-promoting effect of MDSCs from Ad-IL-17 treated B16 tumor-bearing mice was abrogated with administration of anti-*γδ* TCR treatment (Figure 2(e)).

### 3.3. IL-23 Is Critical for the Generation of IL-17

**Figure 3:**
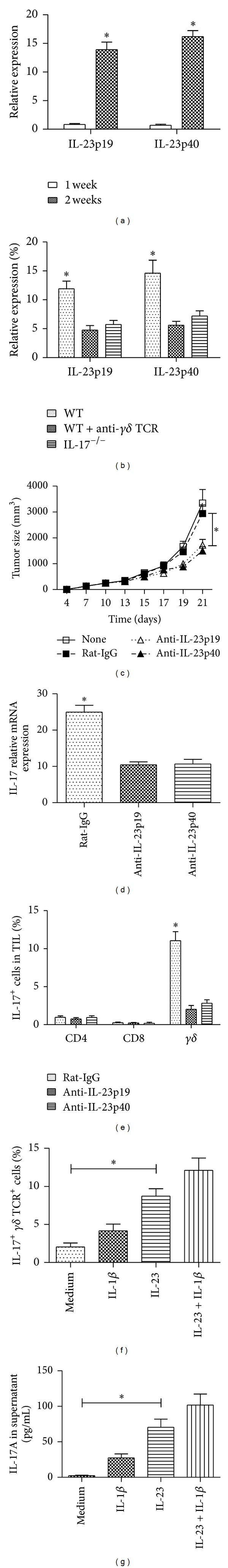
IL-23 is critical for the generation of IL-17 *in vivo* and *in vitro*. (a) IL-23p19 and IL-23p40 mRNA levels were analyzed by realtime PCR in tumor tissues from tumor-bearing mice one or two weeks after B16-F10 inoculation (*n* = 5). (b) IL-23p19 and IL-23p40 mRNA levels were analyzed by realtime PCR in tumor tissues two weeks after B16-F10 inoculation from WT, WT + Anti-*γδ* TCR, and IL-17^−/−^ groups (*n* = 5). (c) Wild-type mice were inoculated with B16-F10 tumor cells and injected i.p. with normal rat-IgG, -IL-23p19 mAb, or -IL-23p40 mAb (100 *μ*g/mouse) on days 0, 1, 6, 10, and 14 (*n* = 5). Then tumor sizes were monitored (*n* = 5). (d) IL-17 relative mRNA expression in tumor tissues from B16-F10 tumor-bearing mice treated with rat-IgG, -IL-23p19 mAb, or -IL-23p40 mAb (*n* = 5). (e) The percentage of cells producing IL-17 in control, anti-IL-23p19, and anti-IL-23p40 treated group (*n* = 5). (f) IL-17-producing *γδ* T cells after stimulation with IL-23. IL-17^+^ *γδ* T cells were analyzed by flow cytometry after stimulation for 48 hours. Tumor lymphocytes were isolated from tumor-bearing mice 1 week after B16-F10 inoculation. The cells were stimulated with medium, IL-1*β* (50 ng/mL), IL-23 (50 ng/mL), or the combination for 48 hours. (g) Increase in the secretion of IL-17 from tumor *γδ* T cells stimulated with IL-23 *in vitro*. *γδ* T cells were purified from tumor lymphocytes by MACS and were stimulated with medium, IL-1*β* (50 ng/mL), IL-23 (50 ng/mL) or the combination for 48 hours. After 48 hours of stimulation, the supernatants were collected, and IL-17 concentrations were measured by ELISA kits. The data show means ± SEM of tumor size and are representative of three independent experiments. *P < 0.05.

To investigate the role of IL-23 in the production of IL-17 by tumor-infiltrating *γδ* T cells, IL-23p19, and IL-23p40, subunits of IL-23 were measured. IL-23p19 and IL-23p40 mRNA expression were significantly increased in tumor tissues 2 weeks after B16-F10 inoculation in wild-type mice (Figure 3(a)). And the expression of IL-23p19 and IL-23p40 mRNA was significantly decreased in anti-*γδ* TCR treated wild-type mice or IL-17^−/−^ mice bearing B16 tumor compared with control group (Figure 3(b)). To further determine whether IL-23 is required for the production of IL-17A, we neutralized its function using an anti-IL-23p19 or anti-IL-23p40 antibody. Melanoma tumor growth was significantly inhibited with treatment of anti-IL-23p19 or anti-IL-23p40 mAb (Figure 3(c)). The number of proliferating cells, the mean blood vessels, and the percentage of CD11b^+^Gr-1^+^ MDSCs were also significantly reduced with anti-IL-23p19 or anti-IL-23p40 mAb administration compared with rat-IgG treatment (Supplementary Figures  3(a)–3(c)). At the same time, IL-17 production was profoundly blockade with treatment of anti-IL-23p19 or anti-IL-23p40 mAb (Figure 3(d)). And the reduced IL-17 production was due to the decreased IL-17-producing *γδ* T cells (Figure 3(e)).

To further confirm the role of IL-23 in the generation of IL-17-producing *γδ* T cells, we stimulated tumor *γδ* T cells with exogenous IL-23 *in vitro*; after stimulation for 48 hours, the percentage of IL-17-producing *γδ* T cells was significantly increased (Figure 3(f)). Supernatant IL-17 from purified *γδ* T cells was increased after stimulation with IL-23, which was further enhanced by IL-23 + IL-1*β* stimulation (Figure 3(g)). Therefore, the *in vitro* experimental data demonstrated that IL-23 is required for the production of IL-17 from *γδ* T cells.

### 3.4. The Hmgb1-RAGE Pathway Mediates the Production of IL-23

**Figure 4:**
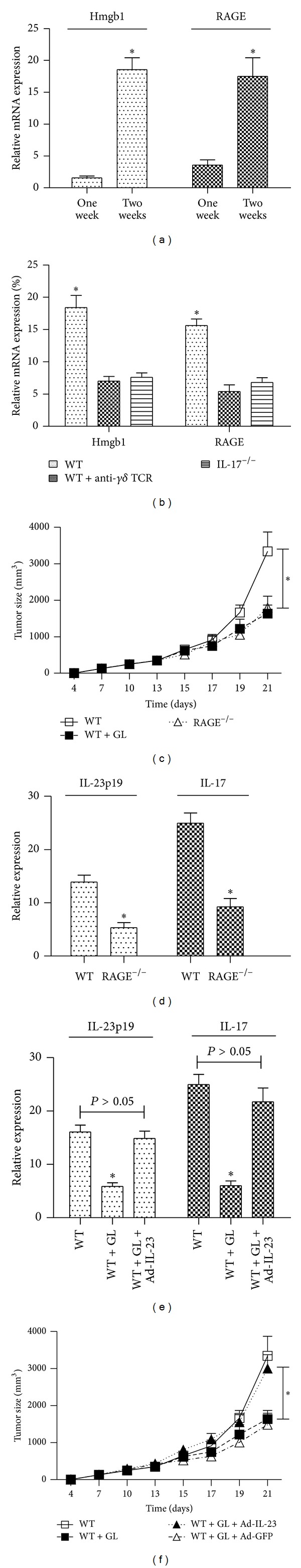
The Hmgb1-RAGE pathway mediates the production of IL-23. (a) mRNA levels of Hmgb1 and RAGE were analyzed by realtime PCR in tumor tissues from tumor-bearing mice one or two weeks after B16-F10 inoculation (*n* = 5). (b) Hmgb1 and RAGE mRNA levels were analyzed by realtime PCR in tumor tissues two weeks after B16-F10 inoculation from WT, WT + anti-*γδ* TCR, and IL-17^−/−^ groups (*n* = 5). (c) Tumor growth in B16 tumor-bearing wild-type mice treated i.p. with 10 mg/mouse glycyrrhizin (GL) on days 0, 2, 5, 10, and 15 and B16 tumor-bearing RAGE^−/−^ mice (*n* = 5). (d) IL-23p19 and IL-17 relative mRNA expression in tumor tissues from B16-F10 tumor-bearing wild-type mice and RAGE^−/−^ mice (*n* = 5). (e) IL-23p19 and IL-17 relative mRNA expression in tumor tissues from B16-F10 tumor-bearing wild-type mice treated with GL or GL + Ad-IL-23 or left untreated (*n* = 5). (f) Tumor growth in B16 tumor-bearing wild-type mice treated with GL, or GL + Ad-IL-23, GL + Ad-GFP or left untreated (*n* = 5). The data show means ± SEM of tumor size and are representative of three independent experiments. *P < 0.05.

Hmgb1, a damage-associated molecule, and its receptor RAGE increased in tumor tissues 2 weeks after B16-F10 inoculation (Figure 4(a)). And the expression of Hmgb1 mRNA was significantly decreased in anti-*γδ* TCR treated wild-type mice or IL-17^−/−^ mice bearing B16 tumor compared with control group (Figure 4(b)). Growth of melanoma cell line B16-F10 was significantly inhibited, and expression of IL-23 and IL-17 was markedly reduced in RAGE^−/−^ mice compared with wild-type mice (Figures 4(c)-4(d)). Use of Hmgb1 inhibitor glycyrrhizin significantly reduced the tumor size (Figure 4(c)) and the production of IL-23 and IL-17, and recombined mouse IL-23 administration abrogated the reduced IL-17 expression and decreased tumor size induced by Hmgb1 inhibitor (Figures 4(e)-4(f)). Furthermore, the number of proliferating cells and mean blood vessels and the percentage of CD11b^+^Gr-1^+^ MDSCs were all markedly decreased in tumor tissues from RAGE^−/−^ mice and glycyrrhizin treated wild-type mice compared with wild-type mice with no treatment (Supplementary Figures  4(a)–4(c)). These findings indicate that Hmgb1-RAGE pathway contributes to IL-17 expression dependent on IL-23 production and then promote tumor growth.

### 3.5. Stat3 Is Activated by IL-17 and Involved in Tumor Development

**Figure 5:**
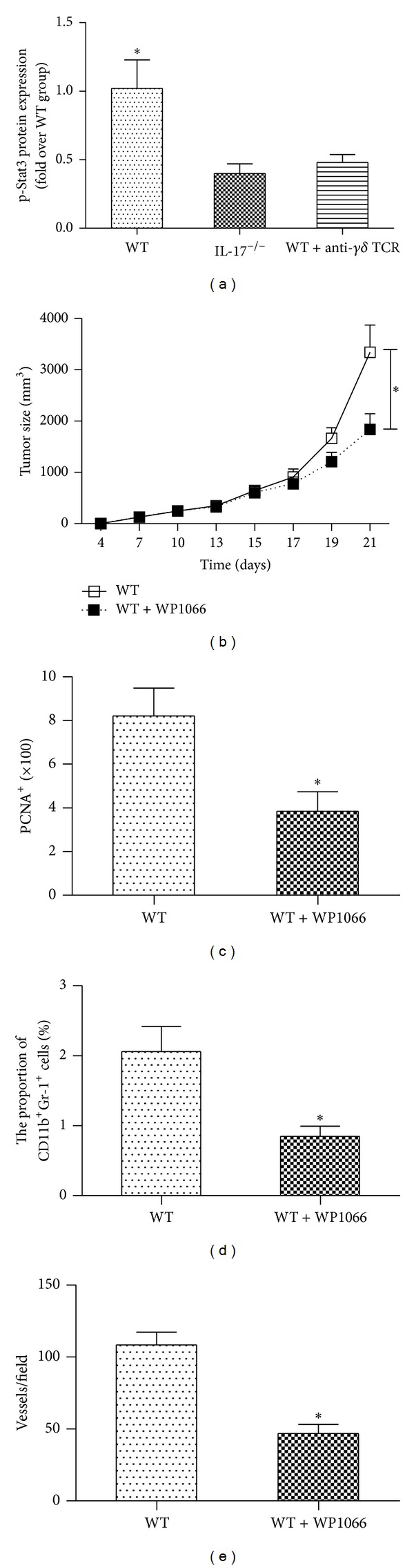
IL-17 promotes Stat3 activation. (a) Indicating p-Stat3 protein levels (analyzed by Western blot) in B16 tumor tissues from WT, WT + anti-*γδ* TCR, and IL-17^−/−^ groups (*n* = 5). (b) Tumor growth in B16 tumor-bearing wild-type mice injected by oral gavage with WP1066 at 40 mg/kg once per day (5 days on and 2 days off) (*n* = 5). (c) The number of PCNA^+^ cells in tumor tissues from B16-F10 tumor-bearing wild-type mice 2 weeks after inoculation (*n* = 5). (d) CD11b^+^Gr-1^+^ MDSCs in tumor samples of B16-F10 tumor-bearing wild-type mice (*n* = 5). (e) The number of blood vessels in tumor tissues from B16-F10 tumor-bearing wild-type mice (*n* = 5). The data show means ± SEM of tumor size and are representative of three independent experiments. *P < 0.05.

Stat3 activation in tumor cells and tumor-associated inflammatory cells plays a critical role in tumor progression by augmenting tumor survival and tumor angiogenesis and suppressing antitumor immunity [31]. Previous paper indicated that IL-17 expression positively correlated with Stat3 activity in growing tumor [13]. In our experiment, Stat3 activity in B16 tumor from wild-type and IL-17^−/−^ mice was examined by immunofluorescence staining of phosphorylated Stat3 (p-Stat3). We found reduced p-Stat3 levels in B16 tumors in IL-17^−/−^ mice and anti-*γδ* TCR treated wild-type mice, as compared with wild-type controls (Figure 5(a)). We next tested whether the Stat3 inhibitor WP1066 shows antitumor activity against B16 tumor cells. We found that the size of B16-F10 inoculated tumor in wild-type mice was significantly reduced with treatment of Stat3 inhibitor WP1066 (Figure 5(b)). The number of proliferating cells and blood vessels and the percentage of CD11b^+^Gr-1^+^ MDSCs were also markedly decreased in tumor tissues from WP1066 treated wild-type mice compared with wild-type mice with no treatment (Figures 5(c)–5(e)).

### 3.6. IL-17 Activates Stat3 through an IL-6-Dependent Mechanism

**Figure 6:**
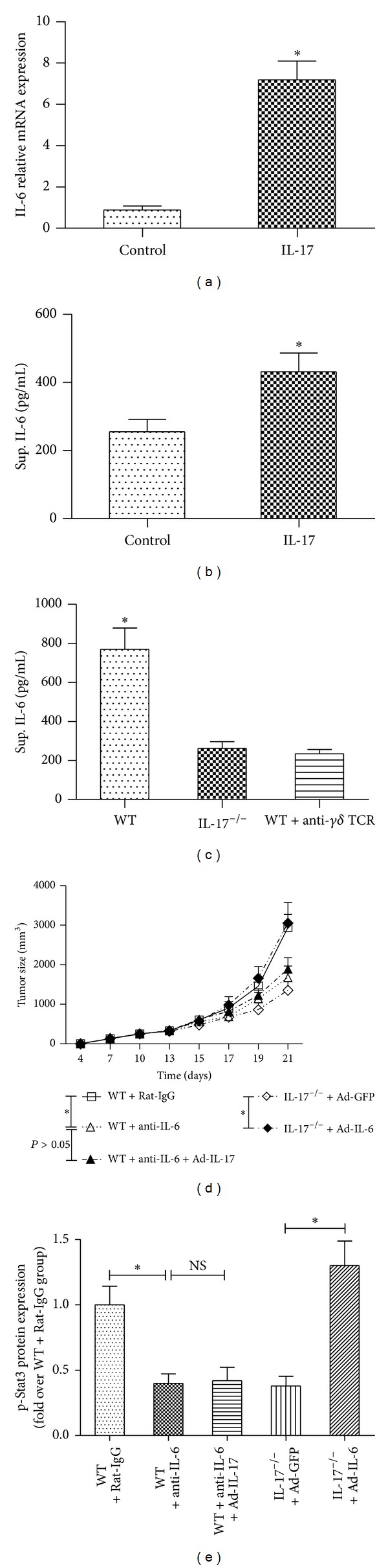
IL-17 activates Stat3 in tumor dependent on IL-6 production. (a) IL-6 level in B16 tumor cells treated for 24 hours with recombinant IL-17 was assessed by quantitative realtime PCR. (b) IL-6 level in B16 tumor cells treated for 24 hours with recombinant IL-17 was assessed by ELISA. (c) Signal-cell suspensions prepared from B16 tumors harvested from WT, WT + anti-*γδ* TCR, and IL-17^−/−^ groups were cultured *in vitro* overnight; supernatants were then collected and assayed for IL-6 levels (*n* = 5). (d) Tumor growth in B16 tumor cells inoculated mice from WT + Rat-IgG, WT + anti-IL-6, WT + anti-IL-6 + Ad-IL-17, IL-17^−/−^ + Ad-GFP, and IL-17^−/−^ + Ad-IL-6 groups (*n* = 5). (e) Indicating p-Stat3 protein levels (analyzed by Western blot) in B16 tumor tissues from WT + Rat-IgG, WT + anti-IL-6, WT + anti-IL-6 + Ad-IL-17, IL-17^−/−^ + Ad-GFP, and IL-17^−/−^ + Ad-IL-6 groups. The data show means ± SEM of tumor size and are representative of three independent experiments. *P < 0.05. NS = not significant.

IL-6 is a Stat3 activator and is elevated in diverse cancers [32]. We therefore determined whether IL-6 mediated IL-17-driven Stat3 activation in a tumor setting. Results showed that IL-17 stimulated IL-6 production by B16 tumor cells *in vitro* (Figures 6(a)-6(b)). We then tested whether IL-17 affected IL-6 production by tumors *in vivo*. Freshly harvested B16 tumor cells from wild-type mice produced relatively high level of IL-6, which was reduced with IL-17 deficiency (Figure 6(c)). In our further study, wild-type mice were challenged with B16 tumor cells, followed by treatment of IL-6-neutralizing antibody or control rat IgG. Results showed that administration of anti-IL-6 mAb but not rat IgG antibody significantly inhibited tumor growth and p-Stat3 expression in wild-type mice. Additional administration of recombinant IL-6 into B16-F10 tumor-bearing IL-17^−/−^ mice resulted in markedly increased tumor size and p-Stat3 expression, whereas additional recombinant IL-17 administration into B16-F10 tumor-bearing wild-type mice treated with anti-IL-6 mAb did not significantly alter the tumor growth and p-Stat3 expression (Figures 6(d)-6(e)).

## 4. Discussion

This study reveals a crucial role for the Hmgb1-IL-23-IL-17-IL-6-Stat3 axis in the development of melanoma. Hmgb1 stimulates the production of IL-23 in a RAGE-dependent manner. IL-23 promotes the expression of IL-17 which is mainly generated by *γδ* T cells. IL-17 then promotes tumor growth through IL-6 induction, which in turn activates Stat3 in tumors.

IL-17 acts as a bridge between adaptive and innate immunities through the potent induction of a gene expression program typical of the inflammatory response, presenting a unique position in the immune response process [9]. Recently, it has been shown that IL-17 is produced by diverse T-cell subsets including CD4^+^ T cells, CD8^+^ T cells, *γδ* T cells, and neutrophils [5]. In our experiment, we found elevated levels of IL-17 in melanoma tumor tissues, and small numbers of CD4^+^ and CD8^+^ T cells producing IL-17, there were much more IL-17-producing *γδ* T cells in tumor tissues. Therefore, our finding provides clear evidence that *γδ* T cells represent a dominant IL-17-producing lymphocyte subset in B16 melanoma.

The role of IL-17 in tumor growth is controversial although it has been intensively investigated, and the mechanism was also not well addressed. Several publications suggested a role of IL-17 in promoting tumor growth [7, 11, 33]. However, recent paper showed that tumors growth was increased in IL-17^−/−^ mice in MC38 sarcoma tumor system [34]. It is possible that IL-17 may have different roles in different tumors and tumor models. In our experiment, we found that IL-17 promotes B16 melanoma growth, whereas the blockade of IL-17 inhibits tumor growth. IL-17 promotes IL-6 induction and Stat3 activation. Treatment of Stat3 inhibitor WP1066 in B16-F10 tumor cells inoculated wild-type mice resulted in reduced proliferating cells, decreased blood vessels, and low percentage of CD11b^+^Gr-1^+^ MDSCs. In our further study, we found that additional administration of recombinant IL-6 into B16-F10 tumor-bearing IL-17^−/−^ mice resulted in markedly increased tumor size and p-Stat3 expression, whereas additional recombinant IL-17 administration into B16-F10 tumor-bearing wild-type mice treated with anti-IL-6 mAb did not significantly alter the tumor growth and p-Stat3 expression. Consistent with our findings, previous paper indicated that IL-17 induces IL-6 production by tumor cells and stromal cells, which in turn activated Stat3 [13]. These data indicate that IL-6 may be a downstream target of IL-17, and IL-17 activates Stat3 in tumor which is IL-6-dependent.

The heterodimeric cytokine IL-23, formed by linkage of the p40 to a p19 subunit, stimulates T-cell differentiation and functions in linking innate and adaptive immunities [35]. IL-23 has emerged as a new player in promoting tumor growth and development [36]. Recent reports indicated that IL-23p19^−/−^ mice were very resistant to skin papillomas, fibrosarcomas, and three different models of experimental tumor metastases [37]. IL-23/IL-17 axis has also been reported to be involved in autoimmunity disease, ischemia-reperfusion injury, and cancers [38–40]. Our results indicate that IL-23p19 and IL-23p40 mRNA expression were significantly increased in melanoma tumor tissues, and depletion of IL-23 resulted in reduced proliferating cells and blood vessels, decreased percentage of CD11b^+^Gr-1^+^ MDSCs, and inhibited IL-17 expression. These data suggest that IL-23 is contributable to the development of melanoma and essential for the generation of IL-17.

Hmgb1, a highly conserved nuclear protein, served as an early mediator of inflammation and cell injury and plays a key role in many pathogenic states including cancers. An elevated expression of Hmgb1 was observed in certain primary tumors including melanoma and colon, prostate, pancreatic, and breast cancers [41]. Mounting evidence showed that the main signaling pathway is activated through the interaction of Hmgb1 and its receptor RAGE. The significance of this pathway *in vivo* was indicated by the observation that blockade of the Hmgb1/RAGE interaction suppressed tumor growth. And the mechanism was associated with diminished IL-6 levels [42]. Our experiment suggests that expression of Hmgb1 and its receptor RAGE was increased in melanoma tumor tissues. Blockade of Hmgb1-RAGE pathway inhibited melanoma tumor growth and reduced production of IL-23 and IL-17. Furthermore, recombined mouse IL-23 administration abrogated the reduced IL-17 expression and decreased tumor size induced by Hmgb1 inhibitor. This indicates that Hmgb1-RAGE pathway contributed to secretion of IL-17 dependent on IL-23 production and then promotes melanoma tumor growth. Report by Kortylewski et al. indicated that IL-23 enhanced the immunosuppressive activity of regulatory T cells within the tumor microenvironment, in part via IL-23-dependent Stat3 activation [43]. Furthermore, recent study by Wild et al. showed that Hmgb1 enhanced inhibitory functions of regulatory T cell via RAGE-mediated mechanisms and limited the number and activity of conventional T cell [44]. All these data suggest that Hmgb1-IL-23-IL-17-IL-6-Stat3 axis plays a pivotal role in melanoma tumor development in a mouse model, and blocking any portion of this axis will attenuate melanoma tumor growth.

In summary, our study provides evidence that Hmgb1-RAGE pathway stimulates the production of IL-23 which promotes expression of IL-17 mainly generated by *γδ* T cells. IL-17 then promotes tumor growth through Stat3 activation dependent on IL-6 induction. Although further investigations are needed to fully clarify the precise molecular and cellular mechanism involved in the immunoregulation, Hmgb1-IL-23-IL-17-IL-6-Stat3 axis contributes to tumor growth in murine models of melanoma.

## Supplementary Material

Supplementary Figure 1: A, The number of PCNA^+^ cells in tumor tissues. Tumor tissues were harvested for analysis 2 weeks after B16-F10 tumor cell inoculation. B, Number of apoptotic cells. C, The number of CD8^+^ T cells. D, The number of blood vessels.
Supplementary Figure 2: A, The number of PCNA^+^ cells in tumor tissues from B16-F10 tumor-bearing mice treated with rat-IgG, -*γδ* TCR or -NKG2D mAb. B, CD11b^+^Gr-1^+^ MDSCs in tumor samples of B16-F10 tumor-bearing mice treated with rat-IgG, -*γδ* TCR or -NKG2D mAb. C, The number of blood vessels in tumor tissues from B16-F10 tumor-bearing mice treated with rat-IgG, -*γδ* TCR or -NKG2D mAb.
Supplementary Figure 3: A, The number of PCNA^+^ cells in tumor tissues from B16-F10 tumor-bearing mice treated with rat-IgG, -IL-23p19 mAb or -IL-23p40 mAb. B, CD11b^+^Gr-1^+^ MDSCs in tumor samples of B16-F10 tumor-bearing mice treated with rat-IgG, -IL-23p19 mAb or -IL-23p40 mAb. C, The number of blood vessels in tumor tissues from B16-F10 tumor-bearing mice treated with rat-IgG, -IL-23p19 mAb or -IL-23p40 mAb.
Supplementary Figure 4: A, The number of PCNA^+^ cells in tumor tissues from B16-F10 tumor-bearing wild-type mice treated with GL or left untreated or from B16-F10 tumor-bearing RAGE^−/−^ mice. B, CD11b^+^Gr-1^+^ MDSCs in tumor samples of B16-F10 tumor-bearing wild-type mice treated with GL or left untreated or from B16-F10 tumor-bearing RAGE^−/−^ mice. C, The number of blood vessels in tumor tissues from B16-F10 tumor-bearing wild-type mice treated with GL or left untreated or from B16-F10 tumor-bearing RAGE^−/−^ mice.

## List of Abbreviations

MDSC: Myeloid-derived suppressor cell
MMP9: Matrix metalloproteinase 9
PCNA: Proliferating cell nuclear Ag
Hmgb: High-mobility group box 1
TLR: Toll-like receptor
RAGE: Receptor for advanced glycation end products
Stat3: Signal transducer and activator of transcription 3
p-Stat3: Phosphorylated Stat3
Bcl-2: B-cell lymphoma-2
TIL: Tumor-infiltrating lymphocytes
GL: Glycyrrhizin.
