# Supplementary material for: Hmgb1-IL-23-IL-17-IL-6-Stat3 Axis Promotes Tumor Growth in Murine Models of Melanoma
Source: Mediators Inflamm. 2013 Dec 28;2013:713859. doi: 10.1155/2013/713859 (PMC3888736; doi:10.1155/2013/713859)
Supplement: Supplementary file 1 — Supplementary Figure 1: A, The number of PCNA+ cells in tumor tissues. Tumor tissues were harvested for analysis 2 weeks after B16-F10 tumor cell inoculation. B, Number of apoptotic cells. C, The number of CD8+ T cells. D, The number of blood vessels. Supplementary Figure 2: A, The number of PCNA+ cells in tumor tissues from B16-F10 tumor-bearing mice treated with rat-IgG, -γδ TCR or -NKG2D mAb. B, CD11b+Gr-1+ MDSCs in tumor samples of B16-F10 tumor-bearing mice treated with rat-IgG, -γδ TCR or -NKG2D mAb. C, The number of blood vessels in tumor tissues from B16-F10 tumor-bearing mice treated with rat-IgG, -γδ TCR or -NKG2D mAb. Supplementary Figure 3: A, The number of PCNA+ cells in tumor tissues from B16-F10 tumor-bearing mice treated with rat-IgG, -IL-23p19 mAb or -IL-23p40 mAb. B, CD11b+Gr-1+ MDSCs in tumor samples of B16-F10 tumor-bearing mice treated with rat-IgG, -IL-23p19 mAb or -IL-23p40 mAb. C, The number of blood vessels in tumor tissues from B16-F10 tumor-bearing mice treated with rat-IgG, -IL-23p19 mAb or -IL-23p40 mAb. Supplementary Figure 4: A, The number of PCNA+ cells in tumor tissues from B16-F10 tumor-bearing wild-type mice treated with GL or left untreated or from B16-F10 tumor-bearing RAGE−/− mice. B, CD11b+Gr-1+ MDSCs in tumor samples of B16-F10 tumor-bearing wild-type mice treated with GL or left untreated or from B16-F10 tumor-bearing RAGE−/− mice. C, The number of blood vessels in tumor tissues from B16-F10 tumor-bearing wild-type mice treated with GL or left untreated or from B16-F10 tumor-bearing RAGE−/− mice. [file 713859.f1.pdf]

Supplementary Figure Legend

Supplementary Figure 1: A, The number of $PCNA^+$ cells in tumor tissues. Tumor tissues were harvested for analysis 2 weeks after B16-F10 tumor cell inoculation. Paraffin sections were prepared and stained with anti-PCNA Abs. PCNA-positive cells were counted microscopically. B, Number of apoptotic cells. Paraffin sections were applied for the TUNEL assay and counterstained with DAPI. Apoptotic cells were counted microscopically. C, The number of $CD8^+$ T cells. Frozen tissue sections were stained with anti-CD8 mAb. The number of $CD8^+$ T cells was counted microscopically. D, The number of blood vessels. Blood vessels were detected by immune-histochemical analysis using cryosections prepared from mice inoculated B16-F10 tumor cells (n = 5). The data show means ± SEM of tumor size and are representative of three independent experiments. *$P<0.05$.

Supplementary Figure 2: A, The number of $PCNA^+$ cells in tumor tissues from B16-F10 tumor-bearing mice treated with rat-IgG, -γδ TCR or -NKG2D mAb (n = 5). B, $CD11b^+Gr\text{-}1^+$ MDSCs in tumor samples of B16-F10 tumor-bearing mice treated with rat-IgG, -γδ TCR or -NKG2D mAb (n = 5). C, The number of blood vessels in tumor tissues from B16-F10 tumor-bearing mice treated with rat-IgG, -γδ TCR or -NKG2D mAb (n = 5). The data show means ± SEM of tumor size and are representative of three independent experiments. *$P<0.05$.

Supplementary Figure 3: A, The number of $PCNA^+$ cells in tumor tissues from B16-F10 tumor-bearing mice treated with rat-IgG, -IL-23p19 mAb or -IL-23p40 mAb (n=5). B, $CD11b^+Gr\text{-}1^+$ MDSCs in tumor samples of B16-F10 tumor-bearing mice treated with rat-IgG, -IL-23p19 mAb or -IL-23p40 mAb (n=5). C, The number of blood vessels in tumor tissues from B16-F10 tumor-bearing mice treated with rat-IgG, -IL-23p19 mAb or -IL-23p40 mAb (n=5). The

data show means ± SEM of tumor size and are representative of three independent experiments. *$P<0.05$.*

Supplementary Figure 4: A, The number of $PCNA^{+}$ cells in tumor tissues from B16-F10 tumor-bearing wild-type mice treated with GL or left untreated or from B16-F10 tumor-bearing $RAGE^{-/-}$ mice (n = 5). B, $CD11b^{+}Gr\text{-}1^{+}$ MDSCs in tumor samples of B16-F10 tumor-bearing wild-type mice treated with GL or left untreated or from B16-F10 tumor-bearing $RAGE^{-/-}$ mice (n = 5). C, The number of blood vessels in tumor tissues from B16-F10 tumor-bearing wild-type mice treated with GL or left untreated or from B16-F10 tumor-bearing $RAGE^{-/-}$ mice (n = 5). The data show means ± SEM of tumor size and are representative of three independent experiments. *$P<0.05$.*

Supplementary figure 1

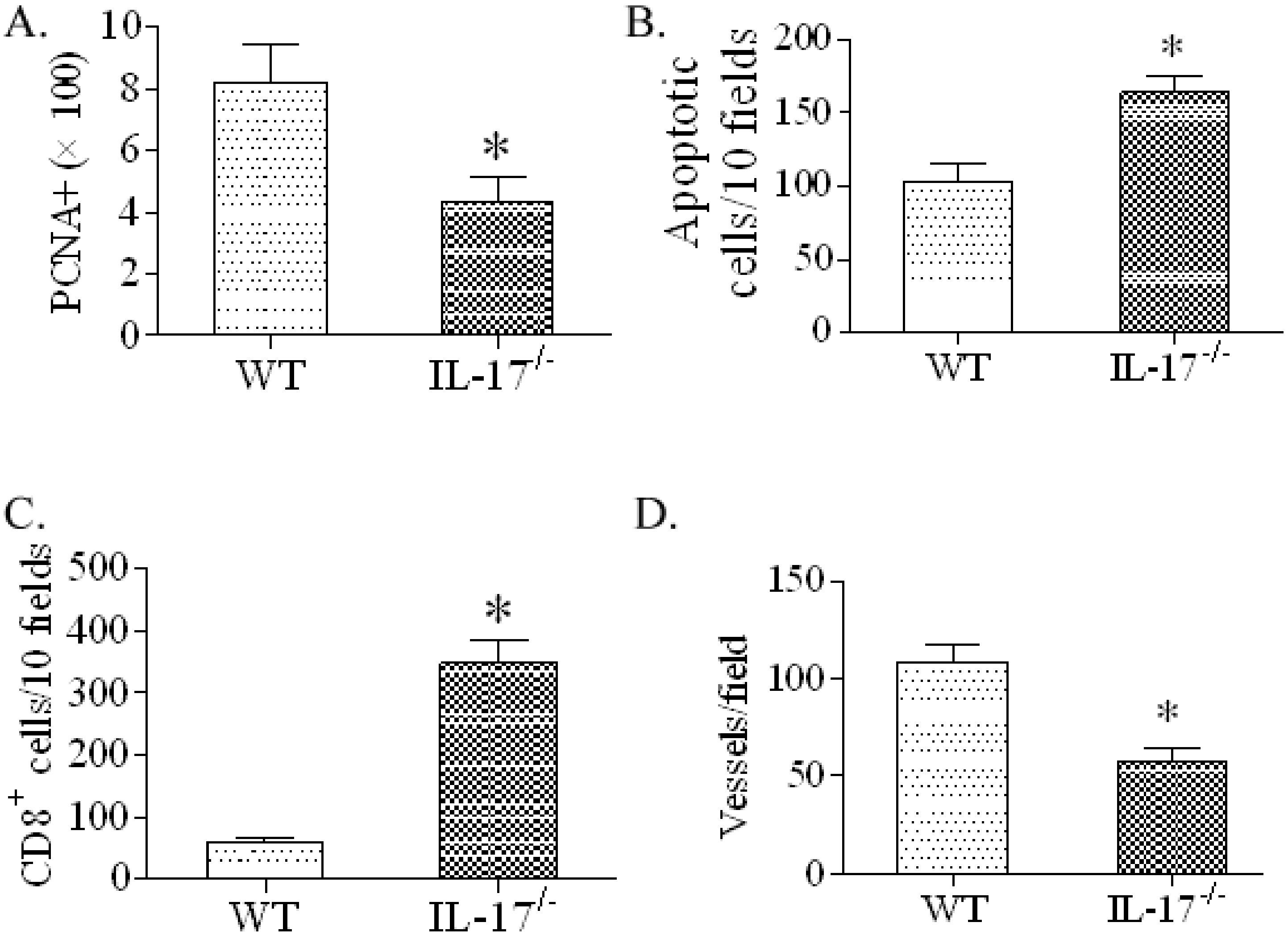

Supplementary figure 2

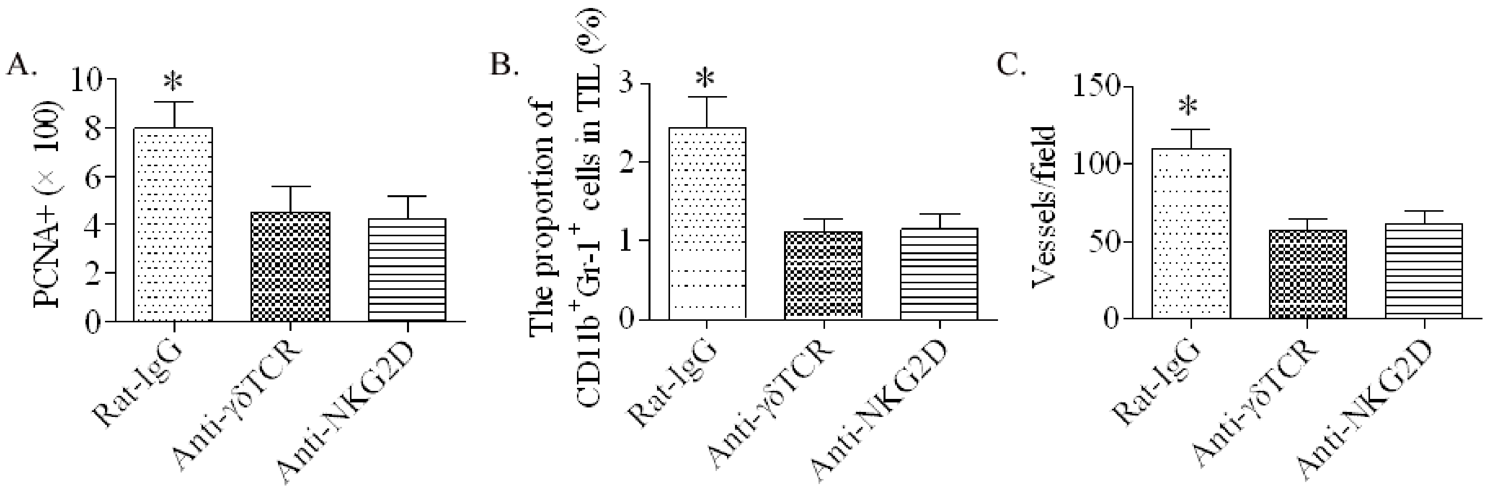

Supplementary figure 3

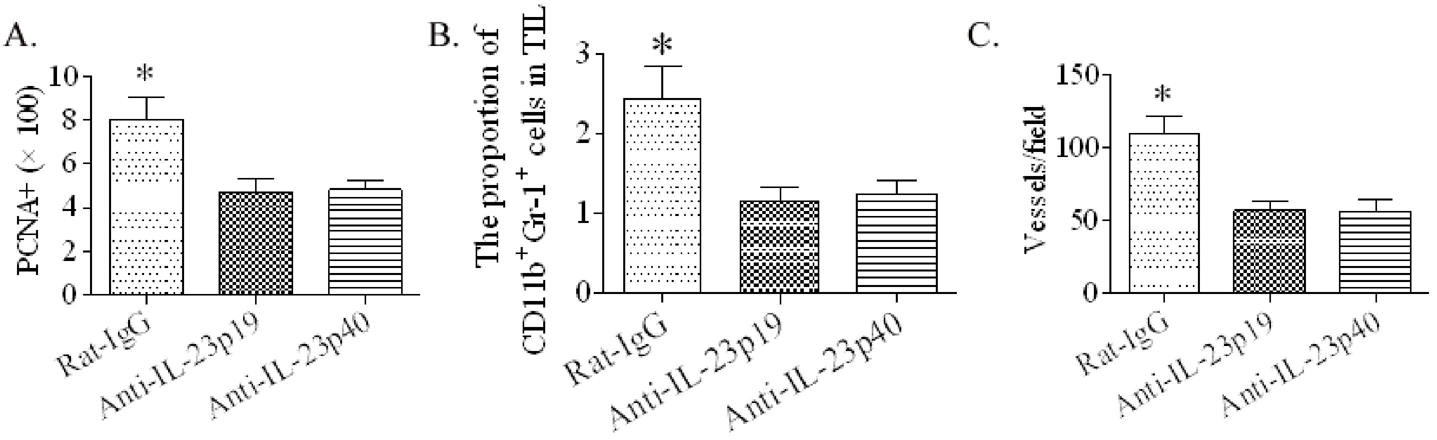

Supplementary figure 4

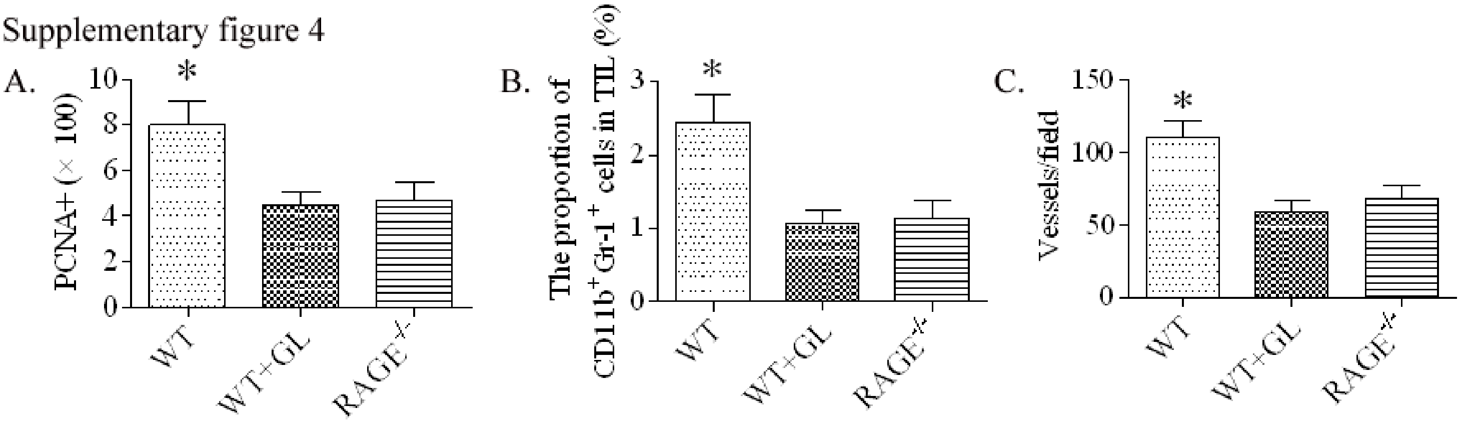

Supplementary Table 1: Primers used for real-time PCR.

Gene	Forward (5'-3')	Reverse (5'-3')
IL-17A	TGTGAAGGTCAACCTCAAAGTCT	GAGGGATATCTATCAGGGTCTTCAT
IL-23p19	AGCGGGACATATGAATCTACTAAGAGA	TCCTAGTAGGGAGGTGTGAAGTTG
IL-23p40	TCCACCAAACTCCCCAGACA	CTGTGCATGCTCTTTGGTTGAT
IL-6	GAGGATACCACTCCCAACAGACC	AAGTGCATCATCGTTGTTCATACA
Arg-1	ATGGAAGAGACCTTCAGCTAC	GCTGTCTTCCCAAGAGTTGGG
MMP9	CATCGAACTTCGACACTGAC	AGCCACGACCATACAGATAC
S100A8	ACAATGCCGTCTGAACTGG	CTCTGCTACTCCTTGTGGCTGTCT
RAGE	CCTGGGTGCTGGTTCTTGCTCT	GATCTGGGTGCTCTTACGGTCC
Hmgb1	ATGGGCAAAGGAGATCCTA	ATTCATCATCATCATCTTCT
β-actin	AAGGCCAACCGTGAAAAGAT	GTGGTACGACCAGAGGCATAC
